# Analysis of Phenolic and Flavonoid Content, *α*-Amylase Inhibitory and Free Radical Scavenging Activities of Some Medicinal Plants

**DOI:** 10.1155/2022/4000707

**Published:** 2022-10-03

**Authors:** Lalit Kala Pandey, Khaga Raj Sharma

**Affiliations:** Central Department of Chemistry, Tribhuvan University, Kirtipur, Kathmandu, Nepal

## Abstract

In Nepal, about 700 plant species have been reported to be used for the primary care of different diseases. However, many of them have not been studied yet for their scientific evidence. The main aim of this study is the quantitative analysis of flavonoids and phenolic content, antioxidant, and antidiabetic activities of the extracts of four different medicinal plants, namely, *Pogostemon benghalensis, Aleuritopteris bicolor, Crateva unilocularis,* and *Rungia pectinata* growing in Nepal. The methanol extracts of plant samples were prepared by the hot percolation method using the Soxhlet apparatus. The phytochemicals of the plant extracts were analysed by colour differentiation methods using different analytical reagents. The phenolic content was estimated by using Folin–Ciocalteu's phenol reagent and the flavonoid was estimated by the aluminium chloride colorimetric method. The 1,1-diphenyl-2-picrylhydrazyl (DPPH) free radical scavenging assay was used to evaluate the antioxidant potential. The *α*-amylase enzyme inhibition activity was performed to evaluate the antidiabetic activity of plant extracts. The amount of total phenolics and flavonoids content was found to be the highest in *Pogostemon benghalensis* (169.43 ± 3.58 mg GAE/g and 65.2 ± 2.0 mg QE/g), respectively, which also showed the most potent free radical scavenging activity (IC_50_ 35.92 ± 0.65 *μ*g/mL). The extract of *Aleuritopteris bicolor* showed the highest *α*-amylase inhibitory activity (IC_50_ 651.58 ± 10.32 *μ*g/mL) whereas *Crateva unilocularis* and *Pogostemon benghalensis* exhibited moderate activity. The extract of *Rungia pectinata* showed the least activity towards *α*-amylase inhibition. Some of the medicinal plants selected in this study showed high TPC and TFC values with potent biological activities. To the best of our knowledge, these medicinal plants have the least exposure to their biological activities, and the results provide scientific evidence for the traditional uses of these plants against diabetes and infectious diseases. However, a detailed study can be performed in these plants to isolate the active chemical compounds and to evaluate *in vivo* pharmacological activities to know the active drug candidates for the future drug development process.

## 1. Introduction

In Nepal people have been using medicinal plants and their products as medicine since the Vedic period. Nepal is a small landlocked country with natural and cultural diversity [1]. In Nepal, it has been reported that more than 700 plant species are to be used for medicinal purposes in the treatment of different human diseases [1–3]. However, a large number of medicinal plants are not explored for their medicinal values and have not yet been studied for their bioactive chemical constituents and potential therapeutic activities. A large number of medicinal plants have been used by ethnic people for the treatment of different diseases, but there is limited information on their mechanism of action, dose frequency, and side effects needed to know the scientific evidence [4, 5]. For this, we selected four traditionally used medicinal plants and analysed their phytochemical constituents with some biological activities.

In plants, the intermediate end products formed by the process of cellular metabolism catalysed by different enzymes are known as metabolites. In plants, primary metabolites are required for growth and development whereas secondary metabolites are not directly involved in growth and development but are necessary for the plant defence mechanism against enemies, environmental, and other cell activities. The secondary metabolites of plants are steroids, terpenes, flavonoids, alkaloids, essential oils, phenolic compounds, and so on. These secondary metabolites play a significant role in the successful treatment of several human diseases contributing to drug discovery either in their original or in the semisynthetic derivative form [6]. Polyphenols are found in most medicinal plants as secondary metabolites with potential anticancer, antioxidant, antimicrobial, anti-inflammatory, and antiallergic activities [7]. The flavonoids act as anticancer, anti-inflammatory, neuroprotective, and cardio-protective in human beings [7]. In the discipline of cancer therapeutic, plant-derived secondary metabolites hold great potential. Triterpenoid saponins and isoflavones play a major role in folk medicine due to their biological and pharmacological properties [8]. The consumption of food enriched with isoflavonoids reduces the risk of various cancer diseases. The vegetables and fruits are rich in flavonoids and polyphenolic substances categorised into flavonols, flavones, flavanones, and biflavones, which play a significant role against different human diseases [9].

*Pogostemon benghalensis* is a flowering plant belonging to the family Lamiaceae. The plant is used as an aphrodisiac, antidepressant, and antibacterial, to treat indigestion, diarrhoea, food poisoning, vomiting, stomach difficulties, cough, cold, typhoid fever, headache, and bodily discomfort. The oil extracted from this plant can be used for skin problems, varicose veins, and haemorrhoids [10].

*Aleuritopteris bicolor* belongs to the family Pteridaceae and likes to thrive in cool, gloomy areas [11]. *Crateva unilocularis* belongs to the family Capparaceae, is widely spread around the world at an altitude of 100–1800 meters and is well-known for its anthelmintic properties. The leaves of this plant are used to treat sinusitis, stomach aches, and hepatitis [12]. *Rungia pectinata* is a weedy plant with many branches and tall stems. The juice from the leaf of this plant is a cooling agent and is used to treat smallpox in children. External application of bruised leaves relieves painful inflammations and swellings. Plant juice may be used to cure cuts and wounds, as well as a decoction used to treat measles and gastrointestinal disorders [13].

The secondary metabolites extracted from the plant parts play a significant role against different infectious diseases, diabetes, cancer, and oxidative stress in the human body. Free radicals in the human body cause oxidative damage to biomolecules, which can lead to numerous diseases, such as atherosclerosis, ageing, cancer, diabetes mellitus, and inflammation. Plants have a wide range of free radical scavenging molecules, such as carotenoids, anthocyanins, flavonoids, carotenoids, and vitamins [14].

Antioxidants are important in shielding biological systems against oxidative stress, which is linked to the development of a variety of chronic illnesses and disorders. Antioxidant supplements can help to mitigate the negative effects of too much ROS (reactive oxygen species) by stimulating the body's defence mechanism. Synthetic antioxidants are important in preventing oxidative stress, which has been linked to the development of neurological illnesses and chronic diseases. Synthetic antioxidants such as butylated hydroxytoluene, butylated hydroxyanisole, and propyl gallate, on the other hand, have been linked to serious adverse side effects. In recent years, the use of natural antioxidants in food has grown in popularity [15–17].

Diabetes mellitus (DM) is a metabolic condition characterized by persistent hyperglycemia and abnormalities in carbohydrate, lipid, and protein metabolism caused by insulin production, insulin action, or both (WHO, 1990). It is a dangerous health disease that's the world's third-highest leading cause of death [18].

The prevalence of diabetes has been increasing globally. Many antidiabetic synthetic drugs are available but the rate of type 2 diabetes is increasing and also these drugs have so many side effects that, have drawn special attention to the necessity of safer, less costly, and more effective management approaches [19]. The *α*-amylase is an enzyme that catalyses the breakdown of starch into sugar. The *α*-amylase hydrolyses dietary starch into trisaccharides and disaccharides, which are then converted to glucose. The *α*-amylase inhibitor is a protein that forms a complex with mammalian *α*-amylase and aids in the management of blood glucose levels [20].

**Figure 1:**
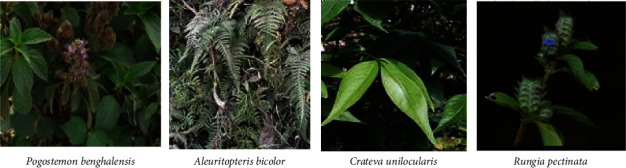
Plant samples used in the study.

The main aims of the present study were focused on the phytochemical analysis, estimation of total phenolic and flavonoid content, *α*-amylase inhibitory activity and the evaluation of the free radical scavenging activity of these four medicinal plants (Figure 1) grown in Nepal. To the best of our knowledge, these plants are not well studied yet for their biological activities along with the determination of total phenolic and flavonoid content.

## 2. Materials and Methods

### 2.1. Chemicals

All the chemicals used in this study were of laboratory grade. The Folin–Ciocalteu's phenol reagent (FCR), *α*-amylase and acarbose were obtained from Sigma Aldrich, St. Louis, MO, USA. The solvent ethanol and methanol (Qualigens Fine Chemicals) were purchased from the local city of Kathmandu, Nepal. Sulphuric acid, hydrochloric acid, aluminium chloride, sodium chloride, sodium carbonate, dimethyl sulphoxide, potassium acetate, disodium hydrogen phosphate dihydrate, and sodium dihydrogen phosphate dihydrate (Thermo-Fisher Scientific India) were purchased from Kathmandu, Nepal. quercetin and gallic acid (Hi-media Laboratories) were procured from the local city of Kathmandu, Nepal. The 1,1-diphenyl-2-picrylhydrazyl (DPPH), gallic acid and quercetin (Wako Pure Chemicals, Osaka, Japan), were purchased from the local city of Kathmandu, Nepal.

### 2.2. Collection and Identification of Plants

The aerial parts of four medicinal plants were collected from different localities in Tadi municipality-4, Nuwakot district of Nepal during the months of March/April 2020. The plants were identified at the National Herbarium and Plant Laboratory, Godawari, Lalitpur, Nepal. The voucher specimens of the plants were submitted to the same department. The voucher specimen number, scientific name, and the local name of the plants are displayed in Table 1.

### 2.3. Extraction

The collected plant parts were washed properly, shade dried, and ground into a fine powder. The powder (100 g) of the plants was kept in the thimble of the Soxhlet apparatus for the hot extraction. After that, the extracts were filtered and concentrated in the rotatory evaporator. The percentage yield of the extracts (Table 2) was calculated using the equation as follows [21]:

(1) $$\begin{array}{c} \text{Yield}\left(\%\right)=\frac{\text{Weight of dried extract}}{\text{Weight of dried plant material}}\times 100. \end{array}$$

### 2.4. Phytochemical Analysis

The plant extracts were subjected to a preliminary analysis to identify the absence or presence of some plants' secondary metabolites using different phytochemical tests [22].

### 2.5. Determination of Total Phenolic Content (TPC)

The total phenolic content in the plant extracts was estimated by using Folin–Ciocalteu's phenol method described by [23] with a slight modification. In brief, 1 mL of plant extract (1 mg/mL) was added to 5 mL of distilled water, and into it 1 mL of Folin–Ciocalteu's phenol was added. The 1 mL of 10% (w/v) Na_2_CO_3_ solution was added and allowed to keep for 1 h. After that, the absorbance was measured at 725 nm using a UV-visible spectrophotometer. The standard calibration curve of gallic acid was constructed and TPC was calculated in mg GAE/g of the extract [24]. All the experiments were performed in triplicates (*n* = 3).

(2) $$\begin{array}{c} C=\frac{cV}{M}, \end{array}$$

where *C* = Total phenolic content in mg.GAE/g, *c* = concentration of gallic acid established from the calibration curve in mg/mL, *V* = volume of extract in mL and *M* = weight of plant extract.

### 2.6. Determination of Total Flavonoid Content (TFC)

The total flavonoid content in the plant's extract was estimated by the aluminium chloride colorimetric method described by Chang et al. [25] with a slight modification. In this method, 1 mL of extract (1 mg/mL) is added to 4 mL of distilled water, followed by the addition of 0.3 mL of 5% sodium nitrite solution. After that, 0.3 mL of 20% aluminium chloride is added and allowed to keep for 6 min. Finally, 2 mL of 1 M sodium hydroxide was added. The absorbance of the resulting mixture solution was measured at 510 nm with the help of a UV-visible spectrophotometer. A calibration curve of quercetin was used to calculate the TFC, which was expressed as mg QE/g of the extract [24]. All the experiments were performed in triplicates. The equation used to calculate the TFC is given below:

(3) $$\begin{array}{c} C=\frac{cV}{M}, \end{array}$$

where *C* = Total flavonoid content compounds in mg GAE/g, *c* = concentration of gallic acid established from the calibration curve in mg/mL, *V* = volume of extract in mL and *M* = weight of plant extract.

### 2.7. Evaluation of Antioxidant Activity

The antioxidant activity of plant extracts was evaluated by a rapid, simple, and inexpensive method using the free radical 1,1-diphenyl-2-picrylhydrazyl (DPPH). The antioxidant activity of the extracts was determined by using a 96-well plate reader with a slight modification of the colorimetric method described by Sabudak et al. [26] and Subedi et al. [27]. The stock solution of plant extracts was prepared by dissolving 1 mg of plant extracts in 1 mL of dimethyl sulfoxide (DMSO). The final concentrations of 10, 25, 50, 75, and 100 *μ*g/mL were prepared by the serial dilution of stock solution in methanol. Ascorbic acid was used as a positive control and methanol as a negative control. The positive control, negative control, and plant extract were loaded at 100 *μ*L of 96 well plate readers in triplicates. Then, 100 *μ*L of DPPH solution was added to each well. Then, it was incubated for 30 minutes in dark. After 30 minutes, absorbance was measured at 517 nm using a UV-visible spectrophotometer.

The scavenging activity of each sample against DPPH free radical was calculated using the equation, scavenging activity (%) = [(Ac−As)/Ac] × 100 [24]. Here, Ac = absorbance of the control and As = absorbance of the sample. The IC_50_ was calculated graphically by plotting the concentration of plant extract against the percentage scavenging. All experiments were performed in triplicates.

### 2.8. Determination of Antidiabetic Activity

#### 2.8.1. *α*-Amylase Inhibition Assay

The *α*-amylase inhibitory activity was studied through the starch iodine procedure with a slight modification [28]. The experiment was carried out in a 96-well microplate in which the final concentrations of 39.06, 78.13, 156.25, 312.5, 625, and 1250 *μ*g/mL solutions were prepared by two-fold dilution using 50% DMSO as solvent. To the total assay mixture, 20 *μ*L enzyme, 20 *μ*L plant extract (acarbose in the case of control), and 10 *μ*L sodium phosphate buffer (pH 6.9 with 6 mM NaCl) were added and incubated at 37°C for 10 minutes. After preincubation, 30 *μ*L of 0.5% starch was added, and it was again incubated at 37°C for 15 minutes. After that, 20 *μ*L of 1 M HCl was added followed by the addition of 100 *μ*L of 1% iodine reagent. The absorbance of the resulting mixture was measured at 620 nm. The control represents 100% enzyme activity without any crude extract (in place of the extract buffer solution, was added to maintain the volume). The percentage inhibition of *α*-amylase was calculated using the following relation.

(4) $$\begin{array}{c} \%\text{Enzyme activity}\left(\text{EA}\right)=\frac{\text{Absorbance starch}-\text{absorbance sample}}{\text{Absorbance starch}-\text{absobance}\left(\text{starch}-\text{enzyme}\right)}\times 100,\\ \text{Inhibition}\left(\%\right)=100-\text{EA.} \end{array}$$

The inhibitory concentration (IC_50_) was calculated graphically, which is the concentration of the sample required for a 50% inhibition of the enzyme. All the experiments were performed in triplicates.

### 2.9. Statistical Analysis

All the experimental data were measured in triplicates (*n* = 3) and the results were presented as mean ± SD. The IC_50_ values were calculated using Graph Pad Prism version 9.0.0 (121). The TPC and TFC were calculated by using Microsoft Excel 2007.

## 3. Results and Discussion

### 3.1. Yields of Plant Extracts

The percentage yield of the plant extracts is shown in (Table 2).

The percentage yield of plant extracts ranges from 6.33% to 17.89%. The highest percentage yield was obtained in *Pogostemon benghalensis* and the lowest in *Rungia pectinata*. The percentage yield of remaining plant extracts was found between these two extremes. The results show the plants are rich sources of secondary metabolites and could be used as the source to isolate the compounds that could be a drug candidate for the future drug development process.

### 3.2. Phytochemical Analysis

The major secondary metabolites present in the plant extracts are shown in Table 3.

The methanolic extract of *Pogostemon benghalensis* showed a positive test for tannins flavonoids, alkaloids, glycosides, polyphenols, reducing sugar, coumarins, saponins, and terpenoids. It has been reported that *Pogostemon benghalensis* showed a positive test for flavonoids, alkaloids, saponins, phenolic compounds, terpenoids, and steroids [29]. Secondary metabolites reported in the extract of *Crateva unilocularis* were alkaloids, flavonoids, tannins and phenolic compounds [30]. Secondary metabolites such as amino acids, glycosides, phenolic, terpenes, phytosterols, tannins, flavonoids, and carbohydrates were reported in *Rungia pectinate* [31].

### 3.3. Total Phenolic and Flavonoid Content

**Figure 2:**
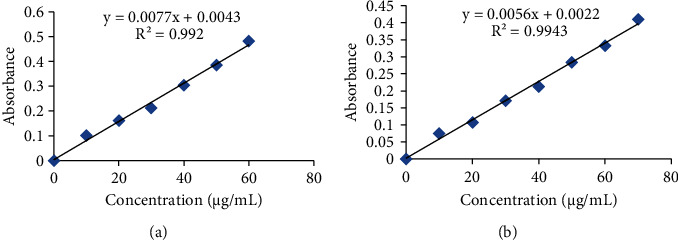
Standard calibration curves of gallic acid and quercetin. (a) Calibration curve of standard gallic acid (b) calibration curve of standard quercetin.

The total phenolic content (TPC) in the plant extract was calculated by using the relationship as (*y* = 0.007*x* + 0.004, *R*^2^ = 0.992). The total flavonoid content (TFC) was also calculated by using the relationship as (*y* = 0.005*x* + 0.002, *R*^2^ = 0.994) shown in Figure 2.

The total phenolic content and flavonoid content was expressed in mg GAE/g of dry extract and mg QE/g of dry extract, respectively. The TPC and TFC of plant extracts are shown in Table 4.

The total phenolic content in the plant extracts ranges from 169.43 ± 3.58 to 105.71 ± 0.57 mg GAE/g dry weight. The plant extract of *Pogostemon benghalensis* showed the highest (169.43 ± 3.58 mg GAE/g) and *Aleuritopteris bicolor* showed the lowest (105.71 ± 0.57 mg GAE/g) TPC among these four medicinal plants. The total phenolic content in the rest of the plant extracts lies in between these two extreme values. In comparing the TFC of four plant extracts, it ranges from 47.87 ± 2.57 to 65.2 ± 2.0 mg QE/g dry weight. The methanolic extract of *Pogostemon benghalensis* showed the highest (65.2 ± 2.0 mg QE/g) and the extract of *Rungia pectinata* showed the lowest (47.87 ± 2.57 mg QE/g) TFC whereas, the other two plant extracts showed a moderate amount of TFC.

### 3.4. Antioxidant Activity

The results of antioxidant activity are shown in Table 5, in which the DPPH radical scavenging potential of plant extracts is expressed as IC_50_ (*μ*g/mL). The radical scavenging activities showed a varying degree of antioxidant property of plant extracts, of which *Pogostemon benghalensis* exhibited the highest percentage of scavenging with an IC_50_ 35.921 ± 0.65 *μ*g/mL followed by *Crateva unilocularis* with IC_50_ value 67.55 ± 0.70 *μ*g/mL. All the plant extracts showed dose-dependent scavenging activity in which the results were compared to the positive control, ascorbic acid (IC_50_ = 17.42 ± 0.30 *μ*g/mL).

**Figure 3:**
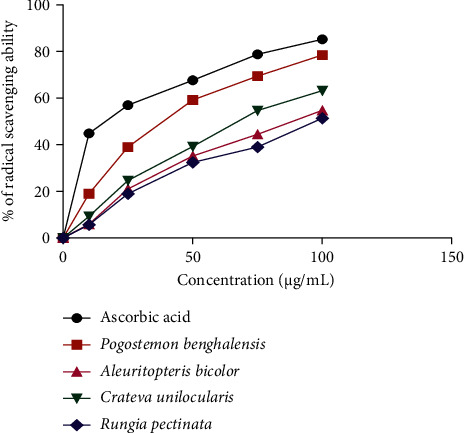
Comparison of percentage radical scavenging of ascorbic acid and plant extracts.

The percentage of radical scavenging against the concentration of plant extracts is shown in Figure 3.

### 3.5. *α*-Amylase Inhibitory Activity

The results of *α*-amylase inhibition activity are shown in Table 6. Among the four tested plant extracts, *Aleuritopteris bicolor* showed moderate *α*-amylase inhibitory activity with IC_50_ 651.58 ± 10.32 *μ*g/mL whereas the *Rungia pectinate* exhibited the lowest inhibitory activity with IC_50_ 1149.98 ± 12.62 *μ*g/mL. The *Aleuritopteris bicolor* could be a good source of natural antidiabetic compounds.

**Figure 4:**
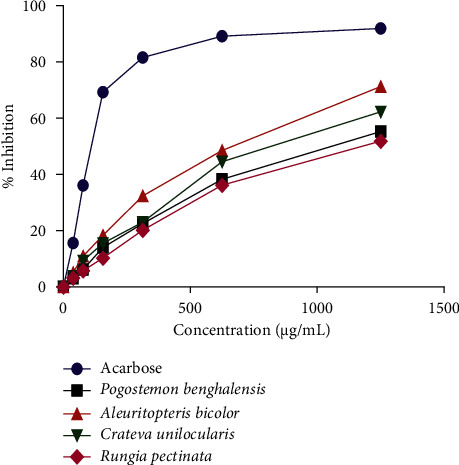
*α*-amylase inhibitory activity of plant extracts and standard acarbose.

The percentage inhibition of *α*-amylase against the concentration of plant extracts is shown in Figure 4.

## 4. Discussion

This study was conducted to evaluate the antioxidant activity, *α*-amylase inhibitory activity, estimation of the total phenolic, and flavonoid content in the four medicinal plants growing in Nepal, but are poorly explored for their biological activities. The results of this study showed that the plants are rich sources of secondary metabolites, rich in total phenolic and flavonoid content, potent natural antioxidants, and moderate towards *α*-amylase inhibitory activities. Phenolic and flavonoid compounds are widely distributed in plants and are included in the human diet, having antioxidant, antidiabetic, and various other medicinal properties. Flavonoids are the largest class of naturally occurring compounds and are reported to have various biological activities [32]. Pandey et al. [33] reported the anthelminthic activity of methanolic extract of *Crateva unilocularis* in *Pheretima posthuma*. The results of the present study showed that plants are good sources of plant secondary metabolites such as alkaloids, saponins, polyphenols, diterpenes, glycosides, coumarins, tannins, flavonoids, and reducing sugar. The total phenolic and flavonoid content were found to be higher in *Pogostemon benghalensis* (169.43 ± 3.58 mg GAE/g and 65.2 ± 2.0 mg QE/g) respectively (Table 4). Phenolic and flavonoid compounds with unsubstituted hydroxyl groups have been recognised as potential free radicals scavengers [34, 35]. The result of DPPH radical scavenging activity showed that the plant extract of *Pogostemon benghalensis* was found to be the potent source of natural antioxidants of IC_50_ 35.92 ± 0.65 *μ*g/mL whereas the rest of the plants were found to have moderate radical scavenging activity. Karle and Saswade [36] reported the antioxidant activity of this plant in five different solvents such as petroleum ether, chloroform, acetone, ethanol, and water. Among these, the ethanol extract exhibited the highest antioxidant activity. The essential oil extracted from the same plant was found active against *the Staphylococcus aureus.* The *α*-amylase inhibitors slow down the hydrolysis of *α*-linked polysaccharides such as starch and glycogen, which ultimately delays the absorption of glucose in blood [37]. In this study, the extract of *Aleuritopteris bicolor* IC_50_ 651.58 ± 10.32 *μ*g/mL showed moderate *α*-amylase inhibition activity. The rest of the plant extracts showed poor inhibition against *α*-amylase.

Results of this study suggested that the plant extracts contain secondary metabolites that are capable of donating hydrogen to the free radicals generated in our body to scavenge the potential damage. Free radicals such as superoxide [38], hydroxyl radicals as well as singlet oxygen species contribute to oxidative stress [39]. The present study correlates the capacity of the phenolic and flavonoid compounds to scavenge the free radical, suggesting its antioxidant potential. It has been reported that phenolic and flavonoid derivatives naturally occurring in plants are thought to have a positive effect on human health. These phenolic and flavonoid derivatives have shown a wide range of antibacterial, antiviral, anti-inflammatory, anticancer, and antiallergic activities [40, 41]. Generally, flavonoid-rich plant extracts scavenge most of the oxidising molecules, including singlet oxygen, and various free radicals, generated in the human body [42].

## 5. Conclusions

In the present study, four medicinal plants growing in Nepal were analysed for their phytochemical constituents, free radical scavenging activity, estimation of total phenolic and flavonoid content, and *α*-amylase enzyme inhibitory activities. From the results of this study, it can be concluded that plant extracts of *Pogostemon benghalensis* and *Crateva unilocularis* were found to possess strong antioxidant activity and a higher content of total phenolics and flavonoids. The *α*-amylase inhibitory activity of *Aleuritopteris bicolor* and *Crateva unilocularis* was found to be higher among the tested plant samples, whereas *Pogostemon benghalensis* and *Rungia pectinata* exhibited moderate activity. These medicinal plants are not well explored for their biological properties, and further studies should focus on the bioassay-guided isolation of chemical compounds and *in vivo* bioactivity evaluations.

## Figures and Tables

**Table 1:** List of plants used in the study, local name, voucher specimen no., and scientific name.

S. N.	Voucher specimen no.	Scientific name (family)	Local name
1.	LKP1 (KATH)	*Pogostemon benghalensis* (Burm. f.) Kuntze	Rudhilo
2.	LKP2 (KATH)	*Aleuritopteris bicolor* (Roxb.) Fraser-Jenk.	Ranisinka
3.	LKP3 (KATH)	*Crateva unilocularis* Buch-Ham.	Siplighan
4.	LKP4 (KATH)	*Rungia pectinata* (L.) Nees	Ukuchi jhar

**Table 2:** The list of plants selected for the study, quantity of extracts and yield values.

Plant samples/extracts	Quantity of sample (g)	Quantity of extract (g)	Colour of extract	Percentage yield
*Pogostemon benghalensis*	100	17.89	Black	17.89
*Aleuritopteris bicolor*	100	11.08	Black	11.08
*Crateva unilocularis*	100	7.79	Yellow	7.79
*Rungia pectinata*	100	6.33	Dark green	6.33

**Table 3:** Phytochemical analysis of the plant extracts.

Group of compounds	*Pogostemon benghalensis*	*Aleuritopteris bicolor*	*Crateva unilocularis*	*Rungia pectinata*
Alkaloids	+	−	+	+
Saponins	+	−	+	-
Polyphenols	+	+	+	+
Diterpenes	+	−	+	-
Glycosides	+	+	+	+
Coumarins	+	+	+	+
Tannins	+	−	+	+
Flavonoids	+	+	+	+
Reducing sugar	+	+	−	+

+ = presence, − = absence.

**Table 4:** The TPC and TFC values of the plant extracts.

Plant samples/extracts	TPC (mg GAE/g)	TFC (mg QE/g)
*Pogostemon benghalensis*	169.43 ± 3.58	65.2 ± 2.0
*Aleuritopteris bicolor*	105.71 ± 0.57	48.27 ± 2.27
*Crateva unilocularis*	143.52 ± 0.87	57.47 ± 1.51
*Rungia pectinata*	111.52 ± 1.57	47.87 ± 2.57

**Table 5:** IC_50_ values against the DPPH free radical scavenging activity of the plant extracts.

Plant samples	IC_50_ for DPPH radical scavenging (*μ*g/mL)
*Pogostemon benghalensis*	35.92 ± 0.65
*Aleuritopteris bicolor*	87.72 ± 2.32
*Crateva unilocularis*	67.55 ± 0.70
*Rungia pectinata*	98.18 ± 2.41
^*∗*^Ascorbic acid	17.42 ± 0.30

Note: values are the mean ± SD (*n* = 3), ^*∗*^ = positive control.

**Table 6:** The IC_50_ values of plant extracts on inhibition of *α*-amylase.

Plant samples	IC_50_ for the *α*-amylase inhibition (*μ*g/mL)
*Pogostemon benghalensis*	1021.09 ± 29.05
*Aleuritopteris bicolor*	651.58 ± 10.32
*Crateva unilocularis*	801.44 ± 6.00
*Rungia pectinata*	1149.98 ± 12.62
^*∗*^Acarbose	107.47 ± 1.38

Note: values are the mean ± SD (*n* = 3), ^*∗*^ = positive control.

## Data Availability

The data used to support the findings of this study are available from the corresponding author upon reasonable request.
